# Bilayer Mucoadhesive Buccal Film for Mucosal Ulcers Treatment: Development, Characterization, and Single Study Case

**DOI:** 10.3390/pharmaceutics12070657

**Published:** 2020-07-11

**Authors:** Thais F. R. Alves, Alesssandra C. Rios, Katiusca da Silva Pontes, Decio L. Portella, Norberto Aranha, Patricia Severino, Eliana B. Souto, Joyce K. M. Gonsalves, Rogeria de Souza Nunes, Marco V. Chaud

**Affiliations:** 1 Laboratory of Biomaterials and Nanotechnology (LaBNUS), University of Sorocaba, Sorocaba 18023-000, Sao Paulo, Brazil; thaisfrancinealves1@gmail.com (T.F.R.A.); alessandra.rios@edu.uniso.br (A.C.R.); katiuscasilvapontes@gmail.com (K.d.S.P.); dportella@pucsp.br (D.L.P.); norberto.aranha@prof.uniso.br (N.A.); 2 Department of Surgery Plastic, School of Medicine, Pontifical Catholic University, Sorocaba 18030-070, Sao Paulo, Brazil; 3 Biotechnological Postgraduate Program, University of Tiradentes (Unit), Aracaju 49010-390, Sergipe, Brazil; patricia_severino@itp.org.br; 4 Nanomedicine and Nanotechnology Laboratory (LNMed), Institute of Technology and Research (ITP), Aracaju 49010-390, Sergipe, Brazil; 5 Faculty of Pharmacy, University of Coimbra, 3000-548 Coimbra, Portugal; ebsouto@ff.uc.pt; 6 Centre of Biological Engineering (CEB), University of Minho, 4710-057 Braga, Portugal; 7 Department of Pharmacy, Federal University of Sergipe, Saint Christopher, Sergipe 49100-000, Brazil; joyce.gonsalves@gmail.com (J.K.M.G.); rogeria.ufs@gmail.com (R.d.S.N.); 8 College of Engineering of Bioprocess and Biotechnology, University of Sorocaba, Sorocaba1 8023-000, Sao Paulo, Brazil

**Keywords:** mucositis, bilayer films, mucoadhesion, biological safety, multidirectional release, single case study

## Abstract

The formation of mucosal ulcers is an end result of epithelial damage, and it occurs due to some specific causes, such as trauma, aphthous stomatitis, lichen planus and lichenoid reactions, cytotoxic effects of chemotherapy and radiation, and drug-induced hypersensitivity reactions and malignant settings. This study focused on films for target drug delivery with respect to the treatment of the diseases of the oral mucosa, specifically mucositis. The results of a single clinical study as a pre-experimental design was performed and followed up to the outcome until 30 days. The polymeric film was prepared in a mucoadhesive bilayer structure: the basal layer with lidocaine HCl had a faster release than the apical layer with benzydamine HCl and *N*-acetyl-cysteine. Fourier Transform Infrared Spectroscopy (FTIR), Differential Scanning Calorimetry (DSC), and SEM characterized the physical–chemical and morphological properties. The cell viability and cytotoxicity were evaluated in cell line MCF7. The transport mechanism of the solvent (swelling) and the drugs in the basal or apical layer (drug release) was explained with mathematical models. To evaluate the effect of movement inside the mouth, the folding endurance was determined. The mucoadhesive bilayer film is biologically safe and stimulates cellular proliferation. A single study in vivo demonstrated the therapeutic effect of the mucoadhesive bilayer film in buccal mucositis.

## 1. Introduction

The formation of mucosal ulcers is an end result of epithelial damage, and it occurs due to some specific causes, such as trauma, aphthous stomatitis, lichen planus and lichenoid reactions, cytotoxic effects of chemotherapy and radiation, and drug-induced hypersensitivity reactions and malignant settings [1,2].

Antineoplastic drugs are cytotoxic; they can induce the formation of intractable ulcerative lesions that provide a primary portal of entry for the secondary infection of buccal flora despite the use of a variety of medicines to prevent them. The mucosal injury progresses from its initiation (1) to a primary damage response, (2) signal amplification, (3) ulceration, and (4) when conditions are favorable for healing [3,4].

The initiation phase is asymptomatic where there is a direct lesion in the DNA of the basal cells of the epithelium and the appearance of oxidative radicals. In the signal amplification phase, the enzymes can be activated directly by radiotherapy and chemotherapy or indirectly by the oxidative radicals formed in the previous phase, inducing tumor cell apoptosis. During the phase of amplification, a series of feedback cycles is observed, further aggravating cellular injury due to the exacerbated production of inflammatory cytokines and the direct destruction of mucosal target cells.

In the ulceration phase, a series of feedback cycles that amplifies mucosal damage is observed, further aggravating the cellular injury. In response to tissue inflammation due to microbial infection and leukocytes infiltration, an exacerbated production of pro-inflammatory and inflammatory cytokines accelerates the destruction of the epithelial cells of the buccal at mucosa and submucosa. However, until the present moment, the precise role of the cytokines has not been clearly defined [5]. This process results in the development of erythematous, atrophic, and ulcerative lesions as a consequence of epithelial damage and death mediated through a complex series of molecular and cellular events that impede the quality of life of individuals with buccal mucositis [2,4,6]. 

The inability to drink, eat, and swallow due to the lymphoedema of the tongue and other parts of the mouth, especially in severe stages, could lead to nutritional deprivation and a serious risk of cachexia due to mucositis [7,8].

The ultimate phase could be the healing, where the processes of epithelial proliferation and differentiation are replaced, and the healthy microflora can re-establish homeostasis in the buccal cavity [3,4,9]. Nevertheless, patients of head and neck cancer rarely reach this stage, even after receiving a moderate dose of radiotherapy [10].

At least 40% and up to 70% of individuals treated with upper-body radiation develop oral mucositis. Few interventions are effective in reducing the duration or severity of mucositis. Regular and systematic buccal hygiene is fundamental to the prevention of mechanical or bacterial injury. The strategies proposed include the use of antimicrobials, antifungals, prostaglandin, radioprotectors, and specific cytokines, including the granulocyte–macrophage colony-stimulating factor, the keratinocyte growth factor, and low-level laser therapy [11,12,13].

The key demands for the treatment of buccal, nasal, esophageal, urethral, vaginal, and perineal rectus mucositis result from the need to prevent or repair and protect the ulcerated mucosa without promoting tumor cell growth. Palifermin is the only drug approved by the Food and Drug Administration (FDA) for mucositis therapy in the U.S. [10]. Mucosal atrophy, weight loss, accelerated mucosal regeneration, and decreased ulceration in patients are all preventively treated with palifermin [14]. Clinical experiments of palifermin in individuals with head and neck cancer demonstrated modest preventive effects on severe buccal mucositis incidence; the median percentage change from baseline to three months was similar between the treatment and the placebo groups, with a reduction of 6.7% and 7.1%, respectively [15].

Administering chemotherapy and radiotherapy to the upper body also gradually affected the salivary gland area during the treatment. The side effects after reducing the amount and quality of saliva produced include xerostomia, thickened saliva, bone pain, nausea, fatigue, sore throat, and tooth decay.

The precarious conditions of the buccal mucosa during the course of chemotherapy and radiation treatment require a smart dosage form that can cover the wound or remain limited to the palate and cheeks. The mucoadhesive buccal films are preferred over mucoadhesive tablets because they are thin and flexible, which induces ease and comfort during routine activities such as eating, drinking, and speaking. Besides that, it can be self-medicated [9,16].

The buccal mucoadhesive films with one or more layers or films based on thermoresponsive hydrogel can be composed by synthetic or natural polymers. In the dosage form, the drug released from the matrix into the oral cavity owing to erosion and is diffusion controlled [17,18,19]. 

Many factors determine the optimum formulation of buccal delivery films, such as the mucoadhesive property of polymers, biodegradation, biocompatibility, drug release, water permeation, physiomechanical properties [18]. The selection of suitable polymers allows the modulation of properties designed for oral films [19,20,21]. The polymers that are mostly used as mucoadhesive are predominantly hydrophilic polymers that swell and allow chain interactions with the mucin molecules in the buccal mucosa [22]. Examples of these swellable polymers include hydroxypropyl cellulose (HPC), hydroxypropyl methylcellulose (HPMC), hydroxyethyl cellulose (HEC), sodium carboxymethyl cellulose (CMC), poly(vinyl pyrrolidone) (PVP), and chitosan (CH) [23].

This study focused on films for target drug delivery to the treatment of illness of buccal mucosa, particularly mucositis. We document and report the results of pre-experimental clinical study design (single type) with follow up to the outcome during 30 days and a single measurement during the sequence of treatment.

The aim was to prepare and characterize bilayer films obtained by the extension of a polymeric gel-like solution, one of them for fast drug delivery (basal layer) and the other for controlled drug delivery (apical layer). The basal layer contains lidocaine. Lidocaine hydrochloride is suitable for reducing pain by blocking the sodium channels of the neurons of local tissues. The apical layer contains benzydamine hydrochloride as a locally acting nonsteroidal anti-inflammatory drug with local anesthetic and analgesic properties and *N*-acetyl-L-cysteine as a salivary antioxidant and fluidizer, which can help relieve symptoms for a variety of diseases exacerbated by xerostomia and reactive oxygen species such as mucositis and the parotid glands damaged by radiation. To our knowledge, there are no other smart bilayer buccal films containing three different drugs for the treatment of buccal mucositis.

## 2. Materials and Methods

### 2.1. Materials

Low molecular weight chitosan (50,000–190,000 Da based on viscosity) with a range of deacetylation 75–95%, viscosity of 20–300 cP 1 wt % in acetic acid 1% *v/v*, at 25 °C) was acquired from Sigma-Aldrich Merck (St. Louis, MO, USA); sodium carboxymethylcellulose was purchased from CPKelco (Limeira, Sao Paulo, Brazil); hydroxypropyl methylcellulose (Methocel K4M^®^) was donated by Colorcon (Cotia, Sao Paulo, Brazil). Lidocaine HCl, benzydamine HCl, and *N*-acetyl-L cysteine were acquired from Sigma-Aldrich Merck (St. Louis, MO, USA). Mucin from porcine stomach type III (sialic acid 0.5–1.5%) was acquired from Sigma Aldrich–Merck (St. Louis, MO, USA). The water was processed to reach resistivity of 18.2 MΩ·cm at 25 °C, and levels below 10 ppb of total organic carbon (Q-Gard Purification Cartridge. Merck KGaA, Darmstadt, Germany). The other reagents were of pharmaceutical grade.

### 2.2. Preparation of Polymeric Gel-Like Solution

Polymeric gel-like solution compositions are shown in Table 1. Formulations F1, F3, and F4 were prepared by dispersing the polymers and other components in purified water. The mixture was homogenized, mechanically stirred at 8500 rpm (T-25D Ultra Turrax disperser, IKA) for 5 min, and left to stand at 10 °C for 24 h or until the total elimination of air bubbles. The F2 formulation containing chitosan (CH) was prepared by dispersing in 1.5 wt % acetic acid and by orbital stirring at 150 rpm, 25 ± 1 °C for 48 h.

For formulations containing benzydamine HCl, *N*-acetyl-L cysteine (F3), and lidocaine HCl (F4), each drug was previously dissolved in purified water and incorporated into the HPMC K4M hydrogel. Then, the F1, F2, and F3 formulations were combined at a ratio of 1.5:4.5:15.0 (wt/wt), respectively, to yield the mucoadhesive polymeric gel-like solution to make film (apical monolayer). The basal monolayer was composed by F4 formulation. The same composition was used to obtain the mucoadhesive polymeric gel-like solution to make the placebo film. At the end, bilayer mucoadhesive films denominated as “A-film” or “P-film” (placebo film) were obtained.

### 2.3. Viscosity of Polymeric Gel-Like Solution

The viscosity of the polymeric gel-like solution was determined using a circulating water bath (Brookfield—TC 550, Massachusetts, MA, USA) configured for use with a digital rotary viscometer (Brookfield—DVI Prime, Massachusetts, MA, USA). A coaxial spindle (SC4-28, Brookfield, Sao Paulo, Brazil) was employed, and viscosity was measured at a constant temperature of 25 °C in a thermostatic bath (TC-550, Brookfield, Sao Paulo, Brazil). Velocity and spindle were previously selected to measure a relative error below 10% and a torque value close to 100%. The recording of the results for viscosity (cP) was done using Wingather 3.0 software. 

### 2.4. Preparation of Mucoadhesive Film in Bilayer

The apical monolayer films were obtained employing an acrylic dispenser with a working volume of 30 cm³ and a slit of 1.0 mm on the lower face, through which the polymeric gel-like solution flowed. The polymeric gel-like solution was dispensed onto a degreased glass plate while the dispenser was moved against it at a constant speed (1.5 cm·s^−1^). The films thus obtained were kept at 23–25 °C in a humid controlled atmosphere (60–70% RH), protected from light and environmental impurities until upon reaching constant weight. Once the apical monolayer films had dried completely, formulation F4 (basal monolayer) was spread on the apical monolayer film using an acrylic dispenser with a working volume at 30 cm^3^ and a slit of 0.5 mm. 

The mucoadhesive films that were removed from the glass plates were cut into 6 cm^2^ pieces (samples) and sterilized by ultraviolet radiation for 3 min. The UV source was kept at a distance of 10 cm from the sample. Then, the sample was tightly sealed in laminated packaging material that was previously sterilized. All manipulation had been done in a biosafety cabinetry Class II Type A2 Certification: NSF/ANSI 49.

### 2.5. Characterization of Mucoadhesive Films

#### 2.5.1. pH Measurement 

The pH of each mucoadhesive film was determined using the pH meter (Tecnal, TEC-5, Piracicaba, Sao Paulo, Brazil). Each sample with 1.0 cm^2^ was dissolved in 10 mL of purified water, which had been previously neutralized. The procedure was performed in triplicate, and the results were recorded.

#### 2.5.2. Measurement of the Uniformity Mass and Thickness of Films

Homogenous circular samples from each mucoadhesive film with a surface area of 6 cm^2^ were cut. The mean weight of five randomly selected samples from each formulation was measured using a highly sensitive microbalance (Semi Microbalance CM 11, Aczet–Atlanta, GA, USA), and the thickness was determined by a digital micrometer (Mitutoyo Digital series-293-832-30–Suzano, Sao Paulo, Brazil).

#### 2.5.3. Folding Endurance Test

Folding endurance tests have been used to estimate the ability of films to withstand repeated bending and folding without any breaking or cracking. The folding endurance tests had been performed in a Stable Micro Systems Texture Analyzer (TA-XT Plus, Godalming Surrey, UK), with analytical probe Mini Tensil Grips and support HDP/FS-R. The folding endurance was measured using Equation (1).

(1) $$FE=\log_{10}N$$

where *FE* is the folding endurance and *N* is the number of double folds required to break the film. The data were registered by the Exponent Connect Lite software when the film breaks.

The test had been observed using ASTM D2176 (American Society for Test and Material). The traveling arm was outfitted with a load cell of 5 kg, and the force response of the sample to the deformation that was imposed on it was recorded. The test followed the current, which was standard ASTM D2176. The sample was clamped in a jaw probe and restrained to moving in a direction perpendicular to the axis of traction without rotating. The load was applied at the traveling arm assembly and adjusted to provide a tension of 1 Kgf on a sample of 6.0 cm^2^. The deflection of the traveling arm was fitted at 2.5 cm.

#### 2.5.4. Swelling Efficiency Profile

The swelling efficiency profile was obtained by the Enslin dipositive [24]. Briefly, the samples were kept in touch with a buffer solution pH of 6.8 for 5 h. This study used films with 1.0 cm^2^ and were put onto the sintering filter, and the volume of media absorbed by the sample after predetermined time was measured with the graduated pipette of the device. The volume of liquid absorbed by the sample was noted and graphically plotted.

#### 2.5.5. Physiomechanical and Mucoadhesive Properties

The TA-XTplus is a Stable Micro Systems’ flagship texture analysis (Vienna Court, Lammas Road Godalming, Surrey GU7 1YL, UK) that is commonly employed to measure and quantify fundamental, empirical, and imitative tests in both compression and tension, covering those relating to texture analysis, materials properties, as well as effects of rheology of solid, semi-solid, viscous liquid, powder and granulate materials. It is coupled with a Peltier-Type Thermoelectric Bath Peltier, a temperature sensor, a circulating pump drain port that was used to maintain temperature accuracy when required, and also an easy-to-use Exponent Connect software; this versatile instrument is extraordinarily well-engineered for long-term reliability and accuracy (Stable Micro Systems, mod. TA-XT plus (Vienna Court, Lammas Road Godalming, Surrey GU7 1YL, UK).

In this study, the TA-XTplus had been used to measure the physiomechanical properties (elasticity, resilience, drilling, and resistance to traction) of mucoadhesive films (A-film and P-film). Before the tests, the calibration of TA-XTplus was done with a load cell of 5 kg. The equipment was outfitted in the compression mode for elasticity, resilience, and drilling, and the traction mode to strengthen the film’s measurements. For the drilling and resistance of traction tests, the velocity of the traveling arm was defined at the rate of 2.0 mm·s^−1^. For elasticity and resilience tests, the velocity of the traveling arm was defined for the rate of 0.5 mm·s^−1^. The elastic (Young’s) modulus was obtained through compression, until the densification of the sample, and it was calculated with a strain that ranged between 0 and 5%.

The mucoadhesive properties were evaluated using a TA-XTplus with mucin discs, and porcine esophageal mucosa was obtained according to the method described by Santana de Freitas-Blanco et al. [25]. Briefly, within 2 h of slaughter, the porcine esophageal mucosa was carefully separated from the surrounding tissue with a scalpel. Mucosa with visible damage at the surface was discarded. After immersion in the purified water at 60 °C for 2.0 min, the epithelium was isolated from the connective tissue and used immediately. Due to its fragility, the epithelium was placed over a pore size of 0.2 μm, diameter of 60 mm, with the connective side of the tissue facing the membrane filter.

Mucin discs were prepared by direct compression (Lemaq, Mini Express LM-D8, Diadema, Brazil) using flat punches, a cylindrical matrix with 8 mm of diameter, and a compression load of 8 tons [26]. The mucin discs showed the planar surface, low porosity, and a diameter and thickness of 8 mm and 0.2 ± 0.01 mm, respectively. Each disc was previously hydrated and fixed to the lower end of the analytical probe-type SC/P10 (TA-XTplus). The samples (6.0 cm^2^) were fixed in the suitable apparatus Mucoadhesion Rig-A/MUC (Stable Micro Systems Texture Analyzer. Godalming Surrey, UK).

The esophageal mucosa covering 1/3 of the lower part of the analytical SC/P10 probe was fixed with a suture line. The mucin disc was fixed in the analytical SC/P10 probe with double-sided adhesive tape. The probe–mucosa and probe–mucin disc were compressed on the surface of the mucoadhesive films with a force of 0.098 N, in the apical to basal direction. In the opposite direction, the probe was detached from the sample surface at 10 mm·s^−1^. Contact times stipulated to evaluate the mucoadhesion between the substrates (mucin or mucosa) and samples were of 45, 90, and 180 s. The force (N) versus distance (mm) profiles provided the peak force, the maximum force of detachment, and the work of adhesion (adhesion energy) as integral to the resultant force–distance profile.

#### 2.5.6. Scanning Electron Microscopy (SEM)

SEM photograph scaffolds were obtained using a scanning electron microscope (LEO Electron Microscopy/Oxford, Leo 440i, Cambridge, UK) with a 10 kV accelerating voltage. The samples were previously dehydrated, quickly frozen in liquid nitrogen, and cut with a circular cutter to prevent damage to the side faces of the films. All the samples were fixed to 12.7 mm pin stubs with a 3.2 mm diameter pin on a multi holder. The films were made electrically conductive by coating them with gold using the sputtering method for 4 min at 15 mA. The pictures were captured in different resolutions. 

#### 2.5.7. Differential Scanning Calorimetry (DSC)

DSC was performed on a TA-60 (Shimadzu, Kyoto, Japan) after being calibrated using indium as the reference material. A sample of 2.0 mg was packed in a hermetically crimped aluminum pan and heated under dry nitrogen flow at 30 mL·min^−1^. The samples were heated from 25 to 200 °C at a rate of 5 °C min^−1^.

#### 2.5.8. Fourier Transform Infrared Spectroscopy (FTIR)

FTIR analysis (Shimadzu, IRAffinity-1, Kyoto, Japan) was used to collect FTIR spectra via Lab Solutions Software v.2.10. The chemical functionalities of the samples were determined by an attenuated total reflectance (ATR) cell on the FTIR spectrophotometer within a range between 4000 and 600 cm^−1^ at 4 cm^−1^ resolutions, averaging 64 scans. 

#### 2.5.9. Drug Content Uniformity

The uniformity of lidocaine HCl, benzydamine HCl, and *N*-acetyl-L-cysteine drugs contents were determined in six sample of A-films, as follows: the mucoadhesive bilayer of the film was cut into slices (6 cm^2^), dissolved in 250 mL of purified water by sonication (40 Hz) for 60 min at 25 °C, and then filtered through a 0.45 µm syringe membrane filter. The lidocaine HCl, benzydamine HCl, and *N*-acetyl-L-cysteine were measured by high-performance liquid chromatography (HPLC). 

The HPLC apparatus used were a Shimadzu LC10AD-VP 9 (Kyoto, Japan), Diode Array Detector SPD-M10AVP, UV-VIS Detector SPD-10A, and Column (00D-4252-EO) Luna C18 (2) 100 Å, with a flow of 1.0 mL/min^−1^. The spine run time was 25 min, and the injection volume was 50 µL. The mobile phase used was acetonitrile/potassium phosphate buffer pH 3.0 (97:3 *v/v*). The detection wavelength was 210 nm, and the column temperature was 25 °C.

#### 2.5.10. Release Profiles of Drugs

An in vitro drug release study was carried out using a type 2 modified dissolution test apparatus (six-station dissolution apparatus). The dissolution medium was prepared with purified water, sodium monophosphate (0.4 wt %), disodium phosphate bi-hydrated (0.4 wt %), sodium bicarbonate (1.38 wt %), and sodium chloride (0.17 wt %) to reach approximately 140 mOsm at pH 6.8. The dissolution medium (250 mL) was maintained at 37 ± 0.5 °C and it was kept in a glass beaker placed inside the dissolution flask. The film (6 cm^2^) was attached to the end of the dissolution tank with a double-sided adhesive tape, which was rotated at 100 rpm. Aliquots of samples (5 mL) were withdrawn at the intervals of 5, 10, 15, 20, 30, 40, 50, and 60 min and filtered through a 0.45 µm syringe membrane filter. The withdrawals were compensated using equal volumes of dissolution medium kept at the same temperature. The concentration of drugs released in the medium at each time was measured as cited above (Section 2.5.9).

The kinetics of the release of drugs were determined to adjust the experimental data to kinetic models. The following mathematical models were used to investigate the kinetics of the release of each drug: zero order, first order, Higuchi, Korsmeyer–Peppas, and Hopfenberg (Table 2). The values of the kinetic parameters and prediction of the drug release were obtained by analyzing the data in an Excel add-on called DD Solver 1.0.

### 2.6. Evaluation of Cytotoxicity

The MCF cells line were maintained in an incubator at 37 °C in an atmosphere containing 5% CO_2_ in bottles with a 75 cm² surface with an inclined neck and filter cap (TPP ™, Waltham, MA, USA) containing 20 mL of DMEM (Dulbecco’s Modified Eagle’s Medium) with phenol red, fetal bovine serum (Nutricell^®^, Campinas, Brazil), penicillin (10,000 IU·mL^−1^), and streptomycin (10 mg·mL^−1^) (Nutricell^®^, Campinas, Brazil). For the periodic repackage—every 72 h, approximately—the whole medium was aspirated and 10 mL of fresh medium was supplemented. With the aid of trypsin, the cells were detached and transferred to new bottles containing the supplemented DMEM medium, as described above. For maintenance of the cell line, 7 × 10^5^ cells·mL^−1^ were seeded by a peal /bottle. All procedures were performed in a laminar flow chamber with prior disinfection of the materials and workbench by ultraviolet (UV) light for 15 min and ethanol 70% *v/v*.

Following the standard of the procedure to isolated cultures, the MCF-7 cells underwent trypsinization, where the cells were detached and centrifuged for the counting technique in the Neubauer chamber. The cells were plated in 6-well cell culture plates (3 × 10^4^ cells). The cells were maintained for 24 h and moved to the survival phase in contact with the membrane. Then, A-film and P-film were placed in duplicates, and 4.5 mL of the culture medium was added. After 24 h, the cells incubated in the presence of films were detached and centrifuged for the counting technique in the Neubauer chamber. The number of cells was recorded by the cells’ viability. 

The contact was maintained for 72 h, and the cytotoxicity levels were evaluated. Following is the evaluation of the influence of the mucoadhesive membrane on MCF-7 cells: the culture medium that was in contact with the cells was removed after 72 h and made DAPI (4′, 6-di-diamidino-2-phenylindole). The blue color of the nucleus was observed through the Nikon TS-100 fluorescence microscope with the attached DXM1200F digital camera. The mucoadhesive films selected for analysis were the bilayer films with and without the addition of drugs. The results of cellular viability were recorded by photographic images.

### 2.7. In Vivo Efficacy of Mucoadhesive A-Film (Single Clinical Case)

The in vivo efficacy of the mucoadhesive film was found by a single case study of an 84-year-old woman under the care of a qualified and trained person. The films were applied after her main meals and after ensuring oral hygiene. The patient and one of her family members signed the “Free and Informed Consent Form” used in clinical practices. All procedures were recognized by the physician responsible for the clinical accompaniment. The patient was taken every three days for clinical evaluation. Participation was voluntary, and the patient and physician could withdraw from the study at any time without giving a reason. The study of the mucoadhesive film application occurred after getting an approval from the Research Ethics Committee of the Santa Lucinda Hospital, Sorocaba, São Paulo, Brazil and College of Medical and Health Sciences of the Pontifical Catholic University of São Paulo. Before the test, the qualified person was oriented toward and trained about the procedure and the objective of the test. The film with 6 cm^2^ was placed on the hard palate, tongue, cheek, or covering the wounds. The patient was asked to avoid using oral antiseptics, antibiotics, or analgesics during the study, as such products might aid in curing the oral ulcers. All other medicines used for the patient for the treatment of chronic illness were under the guidelines.

### 2.8. Statistical Analysis

The main results were presented with their standard deviation. The statistical significance of the difference in the parameters was determined using ANOVA, followed by the Tukey test. A *p*-value of < 0.05 was considered statistically significant.

The main results were presented with their standard deviation. The statistical significance of the difference in the parameters was determined using ANOVA, followed by the Tukey test in GraphPad Prism Version 7.05. A *p*-value of < 0.05 was considered statistically significant.

## 3. Results

### 3.1. Viscosity of Polymeric Gel-Like Solution

The viscosity results of the polymeric gel-like solution are shown in Table 3. The polymeric gel-like blend was prepared to bring together the best properties of each of the polymers as well as to provide synergistic actions among them, ensuring adequate flow behavior for the formation of the film [27].

The high viscosity and film-forming ability of polymers without drugs (P-film monolayer and P-film bilayer) is a result of the interactions between the polymer chains, decreasing the intermolecular spaces and increasing the entanglement. A decrease in the polymeric gel-like blend viscosity was observed when the drugs were added; however, the viscosity still was proper to form films using the appropriated acrylic dispenser [22].

### 3.2. Characterization of Bilayer Mucoadhesive Films

#### 3.2.1. pH, Weight Uniformity, Thickness, and Folding Endurance Measurement

The effect of the different variables on the mucoadhesive films’ properties as pH mass, thickness, and the folding endurance was studied. In a healthy mouth, the drug delivery systems are fit for a pH range of 5.5–7.0 [28]. The pH of film formulations (P-film and A-film) vary between 5.1 ± 0.5. In this pH range, chitosan is dissociated and free to bind with the mucin group electrostatically. The uniformity of the weight and thickness of the films result from the homogeneous mixture, the mass ratio between the polymers, and the chemical compatibility. The weight and thickness of the mucoadhesive films varied from 58 to 79 mg (68.5 ± 10.5 mg) and from 0.11 to 0.13 mm (0.12 ± 0.01 mm), respectively. The log of measurement of the folding endurance Equation (1) was from 2.67 ± 0.07, suggesting that the films have a strength of a minimum of 500 foldings consecutively.

#### 3.2.2. Swelling Efficiency Profile of Bilayer, A-Film (Load Drugs) and P-Film (Placebo)

**Figure 1:**
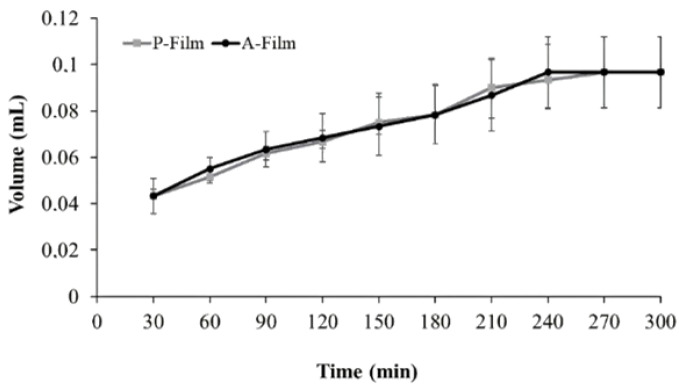
Swelling efficiency profile of bilayer P-film (placebo) and A-film (load drugs) before the start of disintegration.

Figure 1 shows the volume (mL) of buffer absorbed by the sample in the specific time (min). The results show that a hydrophilic equilibrium of the P-film and A-film was reached after 240 min—that is, the curve profile is a baseline until 300 min. The incorporation of drugs to the film’s formulation did not influence the swelling profile (*p* > 0.05).

The absorption of the film profile following the Fickian mathematical model was observed when “*n*” was 0.374 and 0.361, respectively, to P-film and A-film, when the exponent “*n*” is the number that determines the type of diffusion mechanism.

#### 3.2.3. Physicomechanical and Mucoadhesive Properties

**Figure 2:**
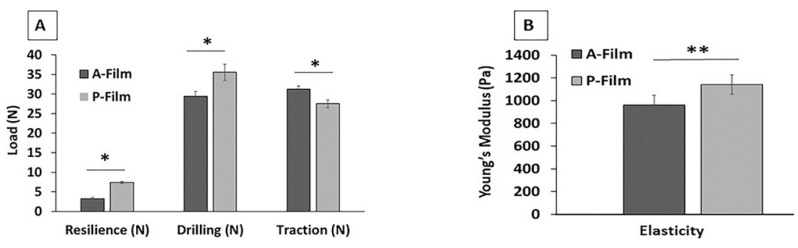
Physiomechanical properties as resilience, drilling, traction (**A**), and elasticity (**B**) of mucoadhesive films. * Means that statistical differences were found between the P-film and A-film (*p* < 0.05). ** Means that statistical equals were not found between the P-film and A-film (*p* > 0.05). (*n* = 3; bar charts represent standard deviation values).

The physicomechanical properties data are shown in Figure 2A,B. Drilling strength, resilience, and Young’s modulus tests measured the film’s ability to compress while resist elongation was conducted in the traction strength test mode. Young’s modulus was measured in the compression mode because the films have a nonlinear elastic stress–strain behavior.

In the presence of drugs in the mucoadhesive films (A-film), the results of resilience, drilling strength, and Young’s modulus (elasticity) were measured over than mucoadhesive films without drugs (P-film) (*p* < 0.05). The resistance to the elongation strength was negatively influenced in the presence of drugs (*p* < 0.05).

**Figure 3:**
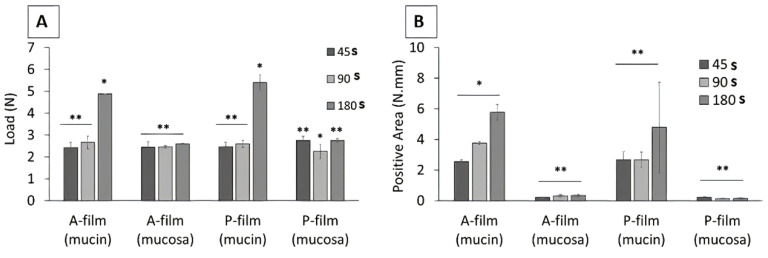
Mucoadhesive properties of mucoadhesive films. The maximum force of detachment (**A**) and the work of adhesion (**B**). * Means that statistical differences were found in different contact times (*p* < 0.05). ** Means that statistical equals were not found in different contact times (*p* > 0.05). (*n* = 3; bar charts represent standard deviation values).

The mucoadhesive properties of the mucoadhesive films were evaluated using mucin discs (in vitro) or porcine esophagus mucosa (ex vivo) at different contact times (45, 90, and 180 s). The mucoadhesive properties of the mucoadhesive films are shown in Figure 3A,B. The force versus distance profiles provided the peak force, the maximum force of detachment (Figure 3A), and the work of adhesion or adhesion energy as integral to the resulting force—distance profile (Figure 3B). 

#### 3.2.4. Morphology of Bilayer A-Film by Scanning Electron Microscopy

**Figure 4:**
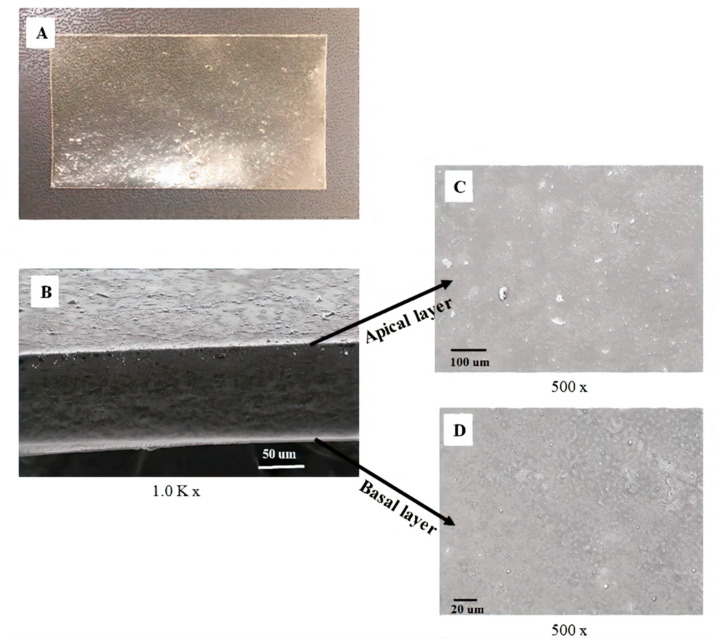
Structure of the bilayer film produced by hydrogel extension. The macroscopic image is observed in panel (**A**). Panels (**B**–**D**) show scanning electron microscopy of the mucoadhesive film (**A**-film) with lidocaine HCl (basal layer), benzydamine HCl, and *N*-acetyl-L-cysteine (apical layer). The sideview image, 1.0K×, shows the bilayer and space between apical and basal layers (**B**). The frontal image 500× shows the apical layer (**C**) and basal layer (**D**).

The morphology of the bilayer film is showed in the micrographs of the cross-section of the A-film, as depicted in Figure 4A, magnification of 1.0 K, and the surface of the apical and basal layers are shown in Figure 4B,C (magnification of 0.5 K). The morphological structure of the film was too thin to be observed by optical microscopy, even with polarized light. However, in the electron microscope, a structure of small insignificant clusters formed during the extension of the hydrogel was revealed. With these samples in the form of semi-thin films, it was possible to obtain a good definition of the space between the basal and apical layers. For resolution higher than 1K, the irregularities observed were probably caused by the loading of the film due to the electron beam. The basal surface is smooth and more uniform than the apical surface, which was deposited on the degreased glass plate surface. The porosity of the plate revealed the small clusters.

#### 3.2.5. Differential Scanning Calorimetry (DSC)

**Figure 5:**
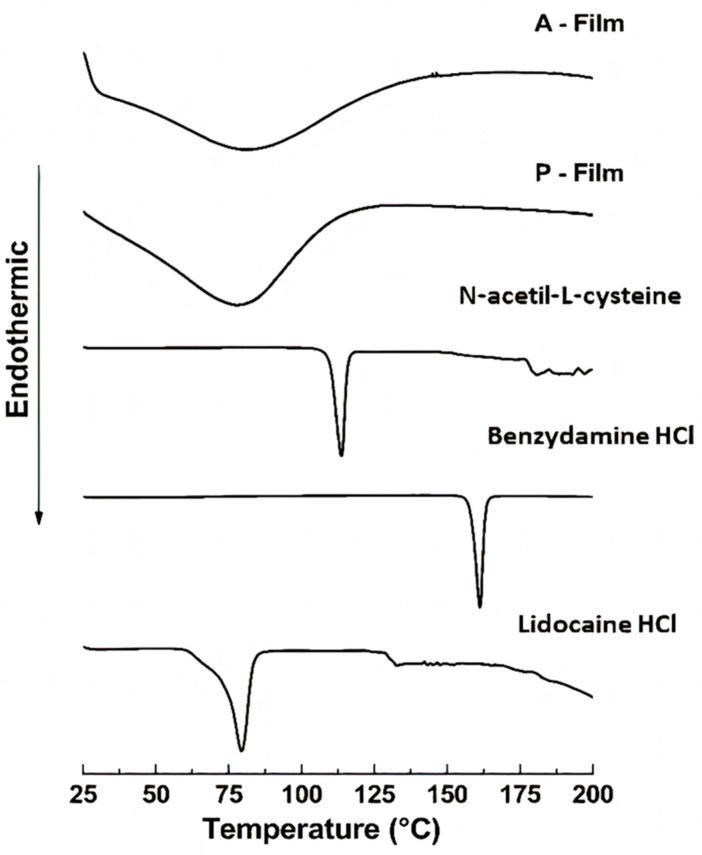
Differential Scanning Calorimetry (DSC) thermograms of A-film with lidocaine HCl, benzydamine HCl, and *N*-acetyl-L-cysteine. P-film without drugs. The curves for pure drug (Lidocaine HCl, Benzydamine HCl, and *N*-acetyl-L-cysteine) are shown.

Representative DSC thermograms of lidocaine HCl, benzydamine HCl, *N*-acetyl-L-cysteine, A-film, and P-film are depicted in Figure 5. The DSC curve of lidocaine HCl showed an endothermic peak at 80.5 °C, corresponding to the melting point of this drug; another thermic event at 130 °C can be related with the glass transition of the material after the fusion of lidocaine HCl. An exothermic event at 177 °C corresponded with thermal degradation [29]. The benzydamine HCl and *N*-acetyl-L-cysteine thermogram show endothermic peaks at 160 °C and 115 °C, respectively, corresponding to the melting point [30,31]. The thermograms of the A-film and P-film show endothermic peaks in the region of the 80 °C, referring to the glass transition temperature of the films and residual water lost.

#### 3.2.6. Fourier Transform Infrared Spectroscopy (FTIR) 

**Figure 6:**
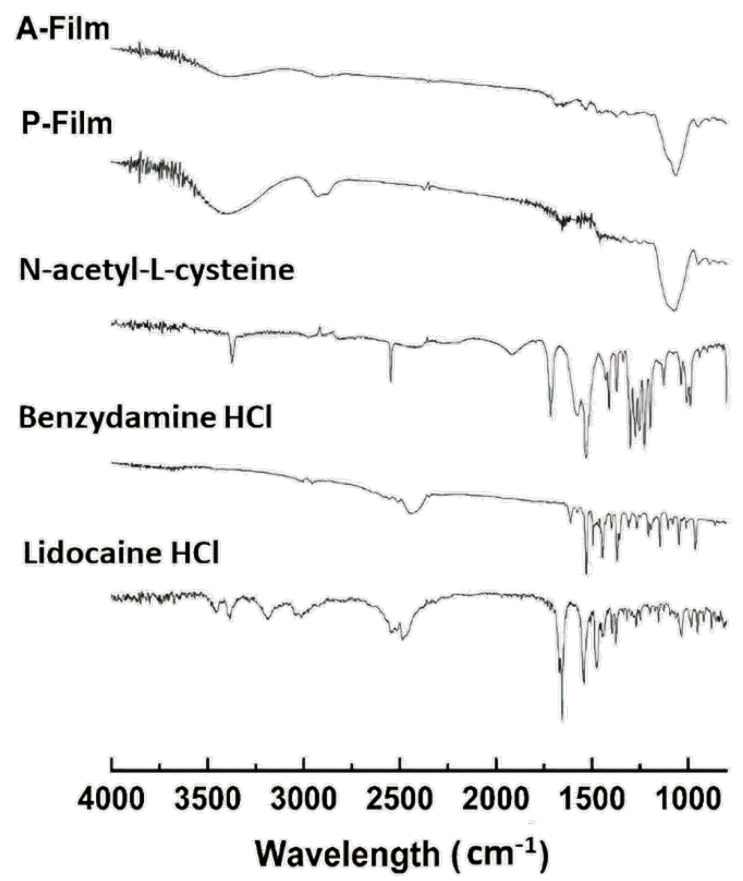
Fourier Transform Infrared Spectroscopy (FTIR) spectra of A-film (with drugs), P-film (without drugs), and pure drugs (Lidocaine HCl, Benzydamine HCl, and *N*-acetyl-L-cysteine) are shown.

The FTIR spectra of drugs and mucoadhesive films are shown in Figure 6. The FTIR spectrum of lidocaine HCl shows O–H stretching and hydrogen bonding intermolecular at 3500 cm^−1^, only alkenes and aromatics show a C–H stretch slightly higher at 3000 cm^−1^, a C–O stretching acid group at 1650 cm^−1^, N–H stretching at 3451, 3385, and 1550 cm^−1^, a C=O stretch carbonyl group at 1475 cm^−1^, and O–H bending at 1250 cm^‒1^ [32]. The typical functional groups of *N*-acetyl-L-cysteine are all well identifiable with full bands in the spectrum. The N–H stretching at 3390 cm^−1^ and the characteristic bands at 2548.5 cm^−1^ are attributed to the stretching of the S–H group. Furthermore, the peaks at 1720 cm^−1^ and 1305.6 cm^−1^ correspond to the C=O group [33], C–N stretching at the 1190–1170 cm^−1^ band, and the N–H stretching vibration is caused due to –NH at 1535.08 cm^−1^. In the FTIR spectrum of benzydamine HCl, the main bands that characterized this drug were identified at 1014 and 2359 cm^−1^ of the C–N stretching and C–O stretching at 1161 cm^−1^ [33].

#### 3.2.7. Drug Content Uniformity

**Figure 7:**
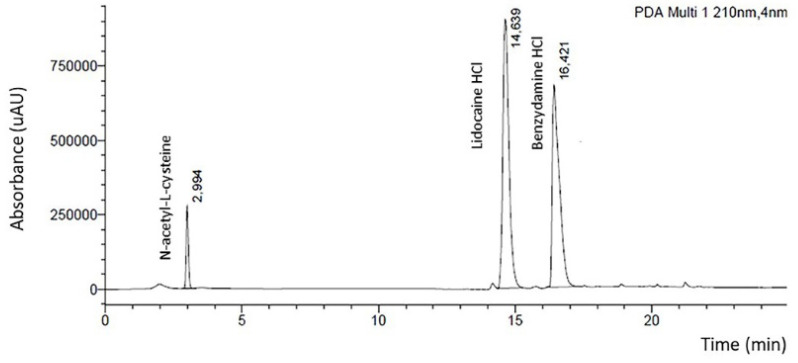
Separation and quantification *N*-acetyl-L-cysteine, lidocaine HCl, and benzydamine HCl by HPLC at 210 nm. The chromatogram representing the specificity of the developed method.

Figure 7 shows typical HPLC chromatograms of *N*-acetyl-L-cysteine, lidocaine HCl, and benzydamine HCl, respectively. The retention time correlating linearly with concentration, correlation coefficient, and regression equation are shown in Table 4. The regression equation had been used to calculate the drug content. The content uniformity (Table 5) of the *N*-acetyl-L-cysteine, lidocaine HCl, and benzydamine HCl in the A-films were 3.59 ± 0.04 mg·mL^−1^, 14.97 ± 1.37 mg·mL^−1^, and 6.69 ± 0.09 mg·mL^−1^, respectively.

#### 3.2.8. Release Profiles of Drugs

**Figure 8:**
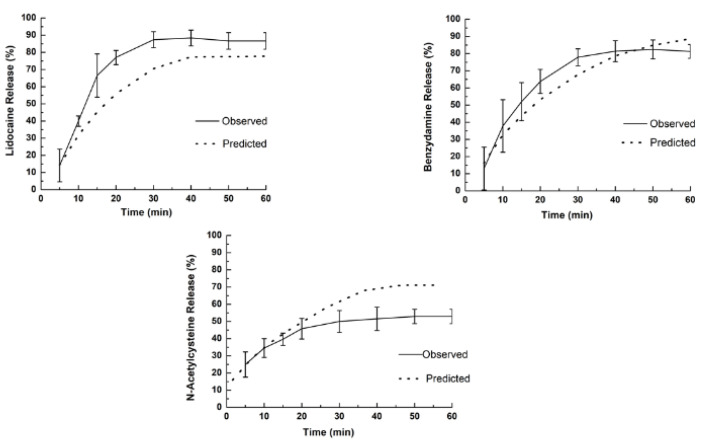
Release profiles observed (solid line) and predicted (dash line) for lidocaine HCl, benzydamine HCl, and *N*-acetyl-L-cysteine from A-film. The dashed lines in the release profile graphs show the values predicted and suitable for better mathematical models (Korsmeyer–Peppas or Hopfenberg).

Dissolution testing is a prerequisite for the optimization of dosage forms; it was designed to follow the pattern described in the main pharmacopoeias under standardized conditions well recognized for temperature, and the composition of the dissolution media, pH, osmolarity, and buffer capacity. The predictive value of an in vitro dissolution profile was calculated using the Hopfenberg mathematic model for lidocaine HCl and benzydamine HCl, and Korsmeyer–Peppas for *N*-acetyl-L-cysteine (Table 6). Both the observed (solid lines) and predicted (dash lines) release profiles are shown in Figure 8.

The composition of dissolution medium previously described contained with purified water, sodium monophosphate (0.4 wt %), disodium phosphate bi-hydrated (0.4 wt %), sodium bicarbonate (1.38 wt %), and sodium chloride (0.17 wt %) to reach approximately 140 mOsm at pH 6.8. Table 5 shows the correlation coefficient of mathematic models tested to explain the release phenomenon of the drugs from A-film. The selection of the appropriate model was in accordance with the required predictive ability and accuracy.

The release profile of lidocaine HCl shows that 85 wt % had been released to 30 min, which was different from the prediction that provided for a maximum release (approximately 70 wt %) in 40 min. The observed release profile for benzydamine HCl was similar to prediction with a maximum of release (approximately 80%) between 40 and 50 min. In the *N*-acetyl-L-cysteine profile, the observed and predicted release was equal in the first 15 min, and it was different for the following times. For the *N*-acetyl-L-cysteine drug, the maximum release after 30 min was approximately 40%.

The mathematical model developed by Hopfenberg has been used to correlate the drug release from surface eroding so long as the surface area remains constant during the disintegration process. Hopfenberg analyzed the release of drugs from surface-eroding devices with several geometries and developed a general mathematical equation describing drug release from slabs, spheres, and infinite cylinders displaying heterogeneous erosion.

### 3.3. Evaluation of Cytotoxicity after 72 h of Contact with A-Film and P-Film

**Figure 9:**
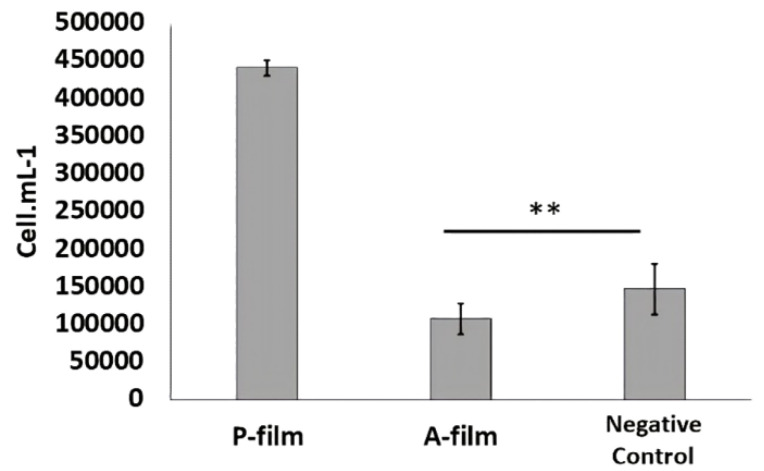
Results of the cell viability study in the presence of P-film and A-film after 24 h (**) no statistical difference (*p* > 0.05). MCF-7 cells were plated (3 × 10^4^ cells/mL^−1^) in a 6-well culture plate and incubated for 24 h. A-film and P-film were placed in duplicated into 4.5 mL of culture medium (Nutricell^®^) and they were incubated for 24 h. The cell growth was measured by counting technique in the Neubauer chamber. The number of cells was recorded and compared with control.

Figure 9 shows the cell viability results in the presence of mucoadhesive films. Previously, the cells have been maintained in culture for 24 h and moved to the survival phase while maintaining contact with A-film and P-film for 72 h. The results show the vitality of MCF-7 cells in the cell culture medium containing the negative control, P-film, and A-film (lidocaine HCl, benzydamine HCl, and *N*-acetyl-L-cysteine). The values represent the number of viable cells per area of the culture region. Although the growth difference is numerically higher for placebo films, addition of the drugs kept the cell count values statistically equal to the negative control.

**Figure 10:**
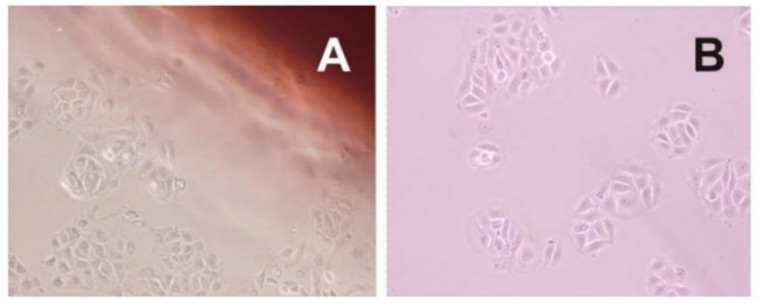
Morphology of MCF-7 cells after 24 h in the presence of P-film (Panel **A**) and A-film (Panel **B**). The micrographics picture shows compact colonies with a typical epithelial polygonal shape of MCF 7 cells. Magnification 40×.

**Figure 11:**
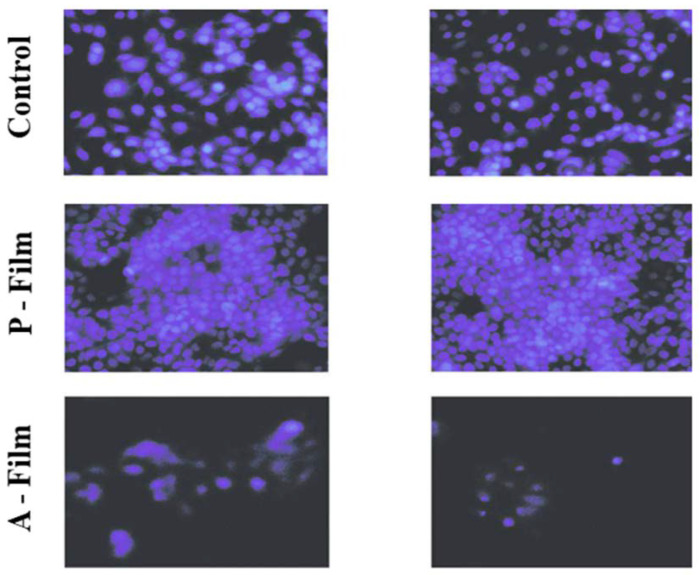
Effect of A-film and P-film on MCF 7 cell viability after 72 h of incubation. The cells density observed by UV-light is higher for P-film, and it is lower for A-film. The blue color of the nucleus treated with 4′, 6-di-diamidino-2-phenylindole (DAPI, 300 nM) was observed and photographed through the Nikon TS-100 fluorescence microscope with the attached DXM1200F digital camera. Magnification 10×.

In Figure 10, the image was captured at 24 h, and the cells are in the process of cell division, forming colonies. Any changes in the morphology of the MCF-7 cell was observed in the presence of P-film and A-film. In Figure 11, the image was captured at 72 h; at this time, the cells were affected by the presence of drugs. However, the results are an indication of biological safety for wound healing.

### 3.4. In Vivo Efficacy of Mucoadhesive Film (Single Clinical Case)

**Figure 12:**
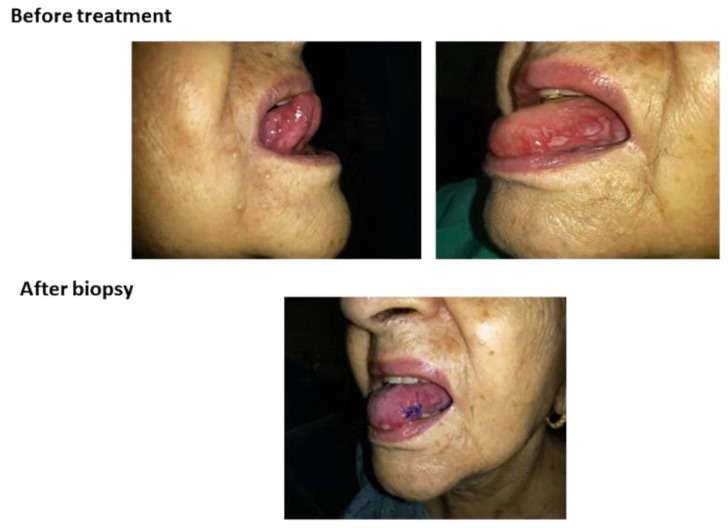
Pictures show lesions in both side of the tongue of the patient before treatment and suture points after a biopsy. The patient is 84 years old, and she had not received chemotherapy/radiotherapy treatment.

Figure 12 shows the picture before treatment and after a biopsy. The pathology report was negative for tumoral cells. The research ethics committee support the case study after the statement of the responsible physician informing that treatments with oral antiseptics, hydrocortisone acetate, neomycin sulphate, troxerutin ointment, and hexamidine spray have not evolved enough for a cure.

**Figure 13:**
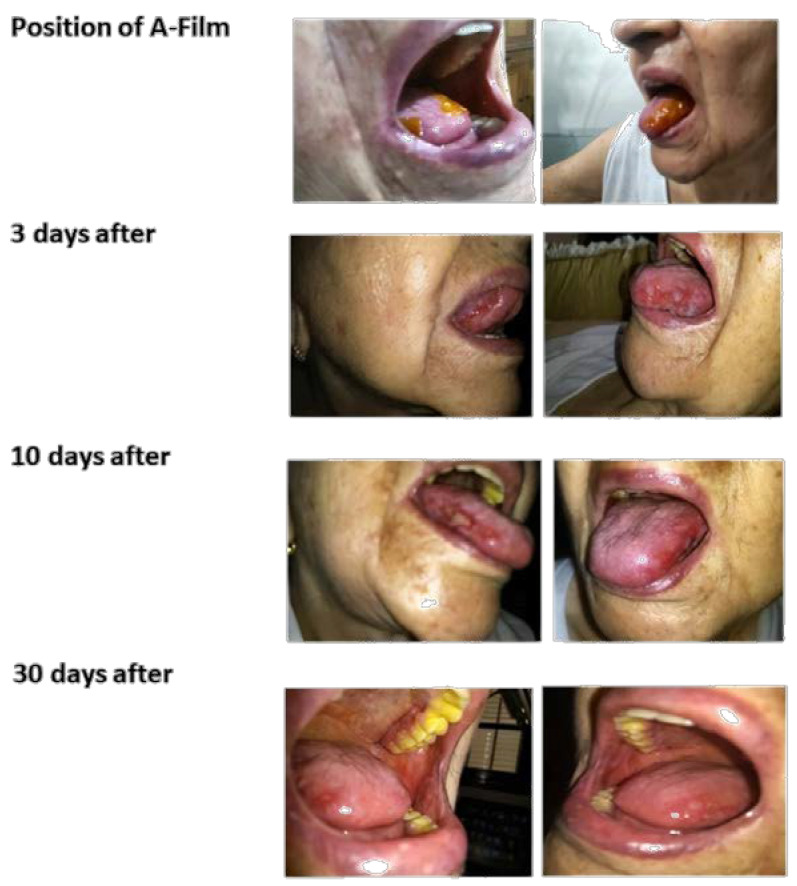
The picture at the top shows the position of the films covering the wounds of the tongue. A film was placed on each cheek and another was placed on the palate. In the sequence, the photos show the mucositis regression course. The cheeks and palate were not photographed because the patient was unable to open her mouth wide enough. On the 30th day of treatment, it is possible to notice the evolution toward healing and a few red spots on the cheeks. The yellow pigmentation on the teeth is from the dye, and it was easily removed in oral hygiene.

Figure 13 shows the picture from the first day to the 30th day before the treatment of the patient with mucositis with the A-film containing lidocaine HCl, benzydamine HCl, and *N*-acetyl-L-cysteine. A significant reduction in ulcer size and the inflammatory process from 10 to 20 days was observed after treatment along with a total regression of mucositis after 30 days. The films that recovered the wound were placed thrice per day on average, and the residence time was 2 h on the tongue, 3 to 4 h on the cheek, and 4 to 6 h under the palate (results not shown). During the treatment, the patient reported having a slightly bitter taste and a decrease in the pain. The increase in the volume of saliva was what caused the greatest discomfort for the patient, which was probably because her parotid is not damaged.

## 4. Discussion

The buccal region has been seen as an advantageous route for drug delivery for the treatment of topical mucosal and submucosal diseases, such as mucositis, periodontitis, gingivitis, and other periodontal and subgingival diseases. However, the thin films for buccal administration have been seen as an alternative approach for the treatment of systemic diseases, whether acute or chronic. The film can be designed for the fast dissolution and immediate release or controlled dissolution and drug release by diffusion or erosion [34]. Fundamentally, thin films are excellent candidates for targeting sensitive sites, which may not be possible with tablets or liquid formulations. Thin films have shown the capability to improve the onset of drug action, reduce the dose frequency, and enhance the drug efficacy [34,35].

The motivation for this work was to note the comorbidities of chemotherapy and radiotherapy for cancer in the upper body, specifically head and neck. In this case, the mucositis and damage of parotid glands are the more severe consequences, and one is the consequence of the other. A severe buccal illness is a cause of loss in the quality life, as it is a bad nutritional condition that can result in cachexia.

The first sign of comorbidity is mucositis, which is responsible for aggressive injuries and the secondary infections in the tongue, cheeks, and the inside of the mouth, causing severe pain and discomfort. The thin film was designed to carry three types of drugs: an anesthetic (lidocaine HCl), anti-inflammatory (benzydamine HCl), and another one with antioxidant and fluidizing activity (*N*-acetyl-L-cysteine).

The strategy for the development of robust dosage form addressed for mucositis treatment and other injuries of oral mucosa was rethinking based on the drug repurposing concepts. Drug repositioning is an effective and highly efficient strategy to find new indications for existing drugs. [36]. So, a comprehensive pre-formulation study has been performed for lidocaine HCl, benzydamine HCl, *N*-acetyl-L-cysteine, and the selection of non-active pharmaceutical ingredients (polymers, dyes, flavoring, and tasting), resulting in two robust pharmaceutical formulas, which could be overlying with one layer on top of another to form a mucoadhesive bilayer film.

The use of polymers and drugs whose physical, chemical properties, and biological behaviors are widely knowledge under stress conditions was at the same time critical to provide a reliable drug repositioning efficient and also laborious, time-consuming, low cost, and riskless.

The main challenge was the release of each drug; the lidocaine HCl should be released, followed by other drugs. To achieve this, a bilayer film was designed, where the basal layer contained lidocaine HCl and the apical layer contained benzydamine HCl and *N*-acetyl-L-cysteine. Then, the basal layer was designed for fast drug release and the apical layer was designed for controlled drugs release.

The other pressing challenge was masking the bitter taste of these drugs and the odor of *N*-acetyl-L-cysteine caused due to the S–H group, because compliance is critical in this case. Hence, sweeteners and flavors were included.

The physiological removal mechanisms in the oral cavity cause mechanical stress and a washing impact by salivation, which can dislodge the drug away from the mucosal site, bringing about a shorter time of exposure and unforeseeable drug distribution at the site of action or absorption [35]. To improve the performance of the bilayer film with the mucoadhesive property, the retention time increased and the wound was covered by linking the chitosan properties.

Mucoadhesive drug delivery systems and multilayer films are assessed as an attractive and feasible dosage form to overcome some negative aspects of conventional local oral dosage forms, such as oral solutions or tablets, without missing the advantage of secure application by patients or their caregivers [17].

During the manufacture of films, great importance was given to the viscosity of polymer dispersion (polymeric gel-like solution), air bubble entrapment, content uniformity, and residual solvents at the final dosage form. The viscosity of the polymeric gel-like solution to be spread will determine the drying rates, reticulation time, and uniformity in terms of the content of the drugs [23]. The polymeric gel-like solution obtained by polymer dispersions with or without drugs showed an appropriate viscosity, which allowed it to be released through a slot with an opening of 0.5 mm (basal layer) or 1.0 mm (apical layer). The bilayer mucoadhesive films showed weight and thickness uniformity, an absence of bubbles, and lower residual moisture (approximately 2.0%).

In the in vitro study (mucin disc), the presence of drugs did not influence (*p* > 0.05) the results for the maximum force of detachment (Figure 3A). However, the contact time showed a positive influence (*p* < 0.05) for 180 s, indicating that the main mucoadhesion theory, in vitro, occurs by electrostatic bend and diffusion–interpenetration of the chain. The work of adhesion or adhesion energy (Figure 3B), in vitro, was positively influenced (*p* < 0.05) for 180 s of the contact. Whereas, for ex vivo study (mucosa), the presence of drugs and the contact time did not influence (*p* > 0.05) the maximum force of detachment (Figure 3A) or work of adhesion or adhesion energy (Figure 3B).

The mucoadhesive films showed a high capacity to resist the folding endurance, which can be attributed to a plasticizer agent (propylene glycol) in a suitable concentration. The interactions of polymer–plasticizer showed a great influence on the film’s flexibility. In general, the polyols impart flexibility to films with increased thickness, allowing easy manipulation of the films without damaging them [1].

Swelling and drug release for a hydro-dispersible matrix can be studied as a simultaneous transport mechanism of liquid into the films and for drug delivery. The amount of liquid determines the swelling rate of a polymeric film that the polymeric matrix can gradually retain. Then, swelling is the consequence of the interaction between a solvent and a matrix [37]. To estimate the swelling rate, the polymers molecular weight and crosslinking bond were designing previously, except for the decision to use the chitosan to increase the mucoadhesion. The swelling rate for A-film was studied and considered useful in understanding the drug delivery mechanism. The greater uptake can be attributed to the higher extent of drug release and for diffusion if total solvation occurs. If not, the mechanism is driven for diffusion and erosion rates.

To understand the swelling mechanism, mathematical models were applied considering the “*n*” at approximately 1. 10^−4^ units, once the magnitude of “*n*” expresses the mechanism transport of the solvent. The results for P-film and A-film were respectively 0.374 and 0.361, which inferred that the swelling rate was slower than the relaxation rate of the polymeric blend crosslinking.

Generally, soft and weak polymers such as CMC, HPMC, and sodium alginate have been describing low tensile strength, low mucoadhesiveness, low elastic (Young’s) modulus, and a low percentage of elongation at break [38,39]. The effects of polyol on the physical properties of biodegradable films have been studied. Results demonstrated that the plasticizer type and concentration influences film mucoadhesiveness, thickness, density, moisture content, solubility, and swelling capacity. Sanyang et al. (2016) showed that gradually increasing the plasticizer concentration decreases the density and water absorption capacity of films; but it increases film thickness, moisture content, and solubility, regardless of the plasticizer type involved. Polyols are osmotically positive compounds, so they reduce the amount of free water [39,40]. In this case, the excess plasticizer could increase the uptake of water of salivary fluid, leading to xerostomia and the thickness of saliva.

The use of sorbitol (sweetener and plasticizer) and propylene glycol (plasticizer) at 1 wt % was found to be an ideal concentration also for mucoadhesiveness, film thickness and resistance to folds, elastic deformation, minimum humidity, swelling capacity and biodesintegration by the erosion of the polymer chains.

The buccal pH contributes to recovering the epithelial formation during the healing phase, where the process of epithelial proliferation and differentiation is renewed and a healthy microflora can be achieved, thereby re-establishing homeostasis [4]. The pH of A-film was 5.1 ± 0.5, which is inferior to values considered normal for a healthy mouth. However, a more acidic pH contributes to the ionization of chitosan, thereby promoting the mucoadhesion by electrostatic interaction with the mucin, forming a polyelectrolyte complex with the mucus; when associated with the entanglement of molecular chains of the mucin and of polymers, new forces of mucoadhesion arise [28].

Texture profile analysis was used to investigate the physiomechanical properties, including mucoadhesion, and mechanical resistance properties. This investigation was also useful in assessing the influence of drugs on the properties of A-film. The basic parameters for designing mucoadhesive films and covering the wound involve other qualities that need analysis during the pre-formulation phase; they include safe and reliable sterilization, a smooth and soft edge, secure handling by individuals without expertise, and easy product application by the patient or by a caregiver.

The lidocaine HCl, benzydamine HCl, and *N*-acetyl-L-cysteine in A-film positively influenced the physiomechanical properties, and the results of the endurance folding test were compatible with Young’s modulus.

The effect of the different variables on the mucoadhesive films’ properties, such as pH mass, thickness, and folding endurance, were studied. The result of the folding resistance test is indicative of the film’s mechanical resistance to the movement of different types of the mucosa and its ability to mold on the surfaces of a specific place of the mouth, such as cheeks, soft or hard palate, and sublingual or vestibule areas. The results obtained for folding endurance are an average of 500 foldings, which were considered safe to support movements of the mouth.

Mucoadhesive polymers can be characterized by testing their adhesion strength by in vitro, ex vivo, and in vivo tests. The mucoadhesive tests for P-film and A-film were performed using in vitro and ex vivo methods of study. The use of the porcine esophagus mucosa changed the mucin adhesion values, showing the importance of simulating the physiological environment for in vitro tests.

The adhesion of films to the oral mucosa is a prerequisite for maintaining drug release. The mucoadhesion of P-film and A-film happens because the chitosan has a positive charge in the formulation pH 5.1 ± 0.5 when interacting with the negatively charged mucin, while hydrated polymeric chains of HPMC 4KM and CMC get entangled with glycoprotein chains of mucin, resulting in higher mucoadhesion. Moreover, HPMC 4KM and CMC can form a gel-like structure at the buccal mucosa, resulting in a larger surface/contact area. This higher surface/contact area increases the water uptake by capillary forces and leads to an increase in the bioadhesive force.

The sum of factors involving electrostatic entangled and contact area positively affected mucoadhesion when compared to studies where at least one of the factors mentioned was absent [9,34,38,39,40].

The diffusion–interpenetration theory describes mucoadhesion involving the interpenetration and entanglement between the polymer chains and the mucus chains. According to this theory, diffusion is dependent on time. The first step in this process involves the creation of initial contact between the bioadhesive polymer chains and the mucus chains, where it involves a weak physical force, e.g., attraction and electrostatic force. The second step involves the interpenetration of polymer chains from the mucoadhesive film into the mucus layer to achieve mucoadhesion via more substantial bond formation [9,39,40].

The morphological appearance of A-film observed in SEM showed continuous sheets with relatively smooth and homogeneous surfaces, suggesting that all the components were uniformly mixed during polymeric gel-like blend formation. Moreover, the uniformity presented in SEM confirm the small standard deviation obtained in the weight uniformity study, physiomechanical properties, and mucoadhesion results.

The DSC analyses showed that the drugs, sweeteners, and flavorings did not result in recrystallization during the process of preparation of the films; that is, the drugs are dispersed in a molecular form, and FTIR can support this result and confirmed the compatibility of components in the film. The FTIR spectra of the films showed the maintenance of characteristic bands of the raw materials. The band around 3500–3000 cm^−1^ shows great intensity due to the stretching of O–H, N–H, and a hydrogen bonding intermolecular force at 3500 cm^−1^ and the associated C–H bond of alkenes and the aromatic groups at 3000 cm^−1^. The changes in the A-film and P-film spectra suggest some kind of interaction between the components, which did not interfere in the design, stability, physiomechanical, and mucoadhesiveness properties of the A-film, as well as in the therapeutic properties of drugs (Figure 13).

The theoretical content of lidocaine HCl, benzydamine HCl, and *N*-acetyl-L-cysteine in each sample of the 6 cm^2^ film are, respectively, 14.7 mg, 8.65 mg, and 4.02 mg. The content of recuperation is shown in Table 5. The loss of 22.4% of benzydamine HCl was considered significant, but the reason was not found. However, it is possible to infer that the loss was by the partial hydrolyses of a functional amide group to pH 6.8. The real concentration (6.69%) had been considered to evaluate the drug delivery profile.

The release profile and dissolution rate of lidocaine HCl, benzydamine HCl, and *N*-acetyl-L-cysteine are shown in Figure 8, where the dashed lines in the release profile graphs show the values predicted and fitted for a better mathematical model (Table 5). As expected, the burst of lidocaine HCl in the basal layer happened as designed, i.e., 15 min before 66.5% of lidocaine was delivered; hence, the objective of the anesthetic effect was achieved in vivo. On the other hand, the release rate of benzydamine HCl and *N*-acetyl-L-cysteine was higher than predicted, and the maximum release rate was achieved with 30 min and 20 min, respectively.

The mathematical model developed by Hoffenberg has been used to correlate the drug release from surface-eroding polymers so long as the surface area remains constant during the disintegration process. Onward, analyzing the release of drugs from surface-eroding devices with several geometries, Hoffenberg developed a general mathematical equation describing the drug release profile from laminated polymeric composite, spheres, and infinite cylinders that displayed heterogeneous erosion [40,41]. The mechanisms by which a drug is released are complex and involve different processes, such as diffusion and erosion. The drug release phenomenon concerning specific mathematical equations revealed information associated with surface properties, liquid uptake behavior, swelling, and erosion of matrix A-film and drug release mechanism [42,43,44].

Fundamentally, the fact that A-film does not fully comply with the Hoffenberg mathematical model can be attributed to the thickness of the film layers and the solubility equilibrium of benzydamine HCl (0.049 mg/mL^−1^) and *N*-acetyl-L-cysteine (23 mg/mL^−1^), which can explain why the result found for the observed release profile was different than that predicted by mathematical models. Therefore, the analysis of release rate showed that the drug delivery was not unidirectional as designed, since the release of three drugs occurred simultaneously, and the in vitro diffusion of the drugs was faster than the erosion of the polymeric chain due to the thin diffusion layer thickness.

The cytotoxicity or cell viability test is one of the essential methods for the safe biological evaluation of biomedical devices such as films, scaffold, implants, and other devices related to the target drug delivery system. The results (Figure 10 and Figure 11) of the MCF-7 cells proliferate and form compact colonies with a typical epithelial polygonal shape. The analysis of cytotoxicity results indicates that P-film and A-film do not affect the cell viability (Figure 9 and Figure 11); hence, the cytotoxicity could be categorized as class 1 (Figure 10 and Figure 11), which demonstrates that the P-films and A-films have cell biocompatibility. Then, this finding was confirmed by a significant reduction of the infectious process, followed by re-epithelization of the lateral surface boards of the tongue (Figure 13). Nevertheless, we are aware that P-film stimulated cell growth in the MCF-7 cell line more than A-film and control (without film). The lowest MCF-7 cell growth for A-film has been attributed to the presence of lidocaine, benzydamine, and *N*-acetyl-L-cysteine, which is an effect that is attributed to the time for cells to adapt to the culture medium.

As a common oral mucosal disease, oral ulcer is characterized by recurrent, painful ulcerations and burning sensations. The majority of mouth ulcers are painful and can adversely affect eating and drinking. During the mucosal injury, an exacerbated production of inflammatory cytokines and the destruction of mucosal target cells are noticed, which cause pain and unease in patients. Moreover, the injury process causes an increase in mucus viscosity, which makes swallowing saliva and food difficult. The buccal mucoadhesive film has been used as an effective dosage form for local oral ulcers. 

The single patient study showed that the association of polymers with the addition of analgesics, anti-inflammatories, and mucolytics promoted the reduction of inflammation, tumefaction, and an erythematous halo with significant mucosal regeneration in 30 days. After 20 days, the patient’s diet changed, she started to eat normally, and due to clinical recommendations, she continued to avoid consuming acidic foods, such as sauces and citrus fruits. The treatment followed the course for another 15 days. After 45 days of treatment, the physician suspended the use of the films and considered the patient cured of mucositis. 

## 5. Conclusions

The mucoadhesive bilayer film designed for the treatment of buccal mucositis achieved the proposed objective. The apical layer with lidocaine HCl and the basal layer with benzydamine HCl and *N*-acetyl-L-cysteine was obtained by a new technique of spreading the polymeric gel-like blend, which facilitated the overlapping of the films and defined the thickness of each layer according to the viscosity and flow of each gel. This process was crucial to achieving physiomechanical properties. Drilling, resilience, resistance on the traction, and the elasticity measurement by Young’s modulus achieved by polymeric formulation in the presence of plasticizer conferred a high folding endurance. The results of physiomechanical and mechanical properties allowed the possibility of estimating the resistance of films to movements of the tongue and cheek. The mucoadhesiveness of the A-film made it possible to estimate the resistance of the films to movements of the tongue and cheek, and above all, covering the wounds without damage to mucoadhesion.

The possibility of covering the wounds was decisive for the treatment of the inflammatory process, reduction of swelling, tissue regeneration, and evolution for healing. The description of symptomatology in the course of the treatment and the clinical evaluation revealed that the careful selection of drugs was correct. Lidocaine HCl fulfilled its local anesthetic effect, successfully blocking the sensation of pain by interfering with the propagation of peripheral nerve impulses. Benzydamine HCl acted in favor of the anti-inflammation and as an analgesic. *N*-acetyl-L-cysteine as an antioxidant and fluidizer of saliva was fundamental for the treatment and regeneration of the mucosa [45]. It is a mucolytic and antioxidant drug that may also influence several inflammatory pathways. It provides the sulfhydryl groups and acts both as a precursor of reduced glutathione and as a direct reactive oxygen species scavenger, hence regulating the redox status in the cells. The preliminary cytotoxicity study showed that the film is biologically safe and stimulated the cellular proliferation. This result was demonstrated in vivo in the single study of the case. However, more in vitro studies are being conducted to evaluate DNA injury before phase II/III clinical studies.

## Figures and Tables

**Table 1:** Composition of polymeric gel-like solution F1, F2, F3, and F4.

Components	F1	F2	F3	F4
Methocel K4M^®^	-	-	3.00	3.00
Sodium carboxymethylcellulose	2.00	-	-	-
Chitosan (low viscosity)	-	3.00	-	-
Acetic acid (1.5%)	-	100	-	-
Sorbitol	-	-	1.00	1.00
Propylene glycol	-	-	1.00	1.00
Hydrogenated castor oil	-	-	0.10	0.10
Sodium chloride	-	-	0.05	0.05
Citric acid	-	-	0.05	0.05
Benzydamine hydrochloride	-	-	1.00	-
*N*-acetyl-L-cysteine	-	-	0.06	-
Lidocaine hydrochloride	-	-	-	2.52
Tartrazine E 102 *	-	-	-	0.0001
Purified water	100	-	100	100

* Tartrazine (E 102), CAS 1934-21-0, is a free water-soluble yellow dye that had been used to identify the basal layer. Sorbitol, propylene glycol, hydrogenated castor oil, sodium chloride, and citric acid had been used as adjuvants to mask the bitter taste of drugs, especially the lidocaine HCl. This adjuvant was firstly dissolved in purified water and after incorporated at each formulation.

**Table 2:** Kinetic models to investigate the kinetics of drugs.

Model	Equation
Zero order	Q_t_ = Q_0_ + K_0_ t
First order	ln Q_t_ = ln Q_0_ + K_1_ t
Higuchi	Q_t_ = K_H_ √t
Korsmeyer–Peppas	Q_t_/Q_0_ = Kk tn
Hopfenberg	Q_t_/Q_0_ = 1 − (1−K_0_t/C_0_a_0_) ^n^

Where Q is the amount of drug dissolved in time t, Q_0_ is the initial amount of drug in the solution (most times, Q_0_ = 0); K, K_0_, K_1_, K_H_, and K_k_ are the release constants; C_0_ is the initial concentration of drug in the matrix; a_0_ is the initial radius for a sphere or cylinder or the half-thickness for a slab; and the n exponent characterizes the release kinetics.

**Table 3:** Measurements of viscosity of polymeric gel-like blend formulations at 25 °C (M ± SD; *n* = 3), for P-Films (placebo F4 formulation, F1 and F4 formulations) and A-films (F1 and F4 formulations).

Polymeric Gel-Like Blend	Viscosity (cP)
P-film (F4 formulation without lidocaine HCl)	4150 ± 188.3 *
A-film (F4 formulation)	3740 ± 225.9 *
P-film (F1–F4 formulation without drugs)	7725 ± 105.2
A-film (F1–F4 formulations)	6758 ± 112.6

* Without statistical difference (*p* > 0.05).

**Table 4:** Results of assay validation parameters of HPLC for the determination of *N*-acetyl-L-cysteine, lidocaine HCl and benzydamine HCl at 210 nm. The concentration (µg/mL^−1^) was linearly correlated.

Parameters	*N*-Acetyl-L-Cysteine	Lidocaine HCl	Benzydamine HCl
Retention time (min)	2.99 ± 0.02	14.63 ± 1.1	16.42 ± 0.04
Concentration (µg/mL^−1^)	1.6–17.6	1.6–19.2	2.7–32.4
Correlation coefficient	0.9973	0.9992	0.9991
Regression equation	y = 7E + 07x − 65577	y = 1E + 08x + 4195.4	y = 2E + 08x − 24180

**Table 5:** The content of drug expected in A-film, real content, and percent of recuperation.

Drugs	Theorical Content (mg) A-Film	Real Content (mg) A-Film	Recuperation (%)
Lidocaine HCl	14.7	14.97 ± 1.37	100
Benzydamine HCl	8.65	6.69 ± 0.09	77.3
N-Acetyl-L Cysteine	4.02	3.59 ± 0.04	100

**Table 6:** Kinetics release of lidocaine HCl, benzydamine HCl, and *N*-acetyl-L-cysteine.

Model	Lidocaine HCl (r^2^)	Benzydamine HCl (r^2^)	*N*-Acetyl-L-Cysteine (r^2^)
Zero Order	0.78398	0.711923	−0.55562
First Order	0.90619	0.936391	0.86619
Higuchi	0.81343	0.870791	0.87079
Korsmeyer–Peppas	0.44038	0.94873	0.94873
Hopfenberg	0.91427	0.96600	−0.53514
